# GC-TOF/MS-Based Metabolomics for Comparison of Volar and Non-Volar Skin Types

**DOI:** 10.3390/metabo12080717

**Published:** 2022-08-03

**Authors:** Ting Bu, Ming Zhang, Sun-Hee Lee, Yu Eun Cheong, Yukyung Park, Kyoung Heon Kim, Dongwon Kim, Sooah Kim

**Affiliations:** 1 Department of Environment Science & Biotechnology, Jeonju University, Jeonju 55069, Korea; buting@jj.ac.kr (T.B.); zhangming@jj.ac.kr (M.Z.); 2 University Provincial Key Laboratory for Protection and Utilization of Longdong Bio-Resources in Gan-Su Province, College of Life Sciences and Technology, Longdong University, Qingyang 745000, China; 3 Department of Biotechnology, Graduate School, Korea University, Seoul 02841, Korea; wowshl@korea.ac.kr (S.-H.L.); yecheong0127@korea.ac.kr (Y.E.C.); khekim@korea.ac.kr (K.H.K.); 4 Graduate School of Energy/Biotechnology, Dongseo University, Busan 47011, Korea; rud8154@gmail.com; 5 Department of Bio-Pharmaceutical Engineering, Dongseo University, Busan 47011, Korea

**Keywords:** GC-TOF/MS, volar skin, non-volar skin, metabolomic analysis

## Abstract

Skin has heterogenous identities on different body sites despite similar cellular compositions. There are two types of skin, volar (palmoplantar) and non-volar (dorsal), which are characterized by epidermal thickness, pigmentation, and presence of hair follicles. However, the mechanisms underlying the development of these different skin types remain unclear. To investigate these, we profiled the cellular metabolites of volar and non-volar skin in mice using gas chromatography-time-of-flight/mass spectrometry (GC-TOF/MS), and further assessed the metabolic differences between them. In total, 96 metabolites from both volar and non-volar skin of mice were identified using the BinBase database system. Metabolomics analysis revealed important differences associated with amino acid metabolism (phenylalanine, tyrosine, and tryptophan biosynthesis; aspartate and glutamate metabolism), sugar metabolism (pentose phosphate pathway), and nucleotide metabolism (pyrimidine metabolism) in volar skin. Fifty metabolites were identified as potential biomarkers differentiating the physiological characteristics of these skin types. Of these, nine were highly increased whereas 41 were significantly decreased in volar skin compared with those in non-volar skin. Overall, these results provide valuable information for understanding the metabolic differences between volar and non-volar skin.

## 1. Introduction

Despite similar cellular components, human skin shows remarkable anatomic diversity in its functions across various body sites. From a fundamental scientific perspective, the anatomic diversity of human skin raises interesting questions regarding how cells obtain and maintain their site-specific identities in self-renewing tissues. Indeed, dermis tissue has two subpopulations of fibroblasts with different physiological functions. Papillary fibroblasts, located in the upper dermis and close to the epidermis [1,2], successfully induce new hair follicle regeneration and wound healing, compared with the reticular fibroblasts spread throughout the lower dermis [3,4]. This fibroblast heterogeneity may depend on the composition of the extracellular matrix (ECM) and factors secreted from different dermal locations [1]. The epidermis comprises distinct layers with keratinocytes of different status, resulting in epidermal stratification. Chang et al. used cDNA arrays and demonstrated that the gene expression patterns in human dermal fibroblasts from different anatomical sites are significantly different [5,6].

Epidermal development in mammals involves multiple processes, including epidermal specification, stratification, differentiation, and growth, and is closely associated with development of the dermis and mesenchyme. Furthermore, epidermal stratification is regulated by the contribution of site-specific dermal fibroblasts [7,8]. In mammals, volar (palmoplantar) skin (i.e., the palm and sole) is characterized by the increased epidermal thickness, less pigmentation, and lack of hair follicles compared with that in non-volar (dorsal) skin (i.e., back, trunk, and ears) [9]. Previous transcriptomics studies have demonstrated that cytoskeletal KERATIN 9 (KRT9) is exclusively expressed in volar epidermis [10,11]. Further, its expression is induced by volar fibroblasts and is regulated by WNT/β-catenin signaling [12,13], suggesting that epidermal morphogenesis and homeostasis are maintained by extrinsic factors from fibroblasts [14,15,16]. However, the mechanisms by which different identities of volar and non-volar skin are established and maintained remain unclear.

Metabolomics involves the analysis of metabolites and metabolic pathways in cells. Compared with other omics technologies, such as genomics, transcriptomics, and proteomics, metabolomics is considered to offer valuable information that represents the phenotypic changes occurring in living organisms because of the acquired environment [17,18,19]. Especially, GC/MS has proven to be a potentially useful platform for metabolic analysis because of its well-established database, reliability, reproducibility, and ease of use [20,21,22]. Similar to other tissues, skin contains numerous metabolites secreted from sweat; this broad spectrum of metabolites may offer new biomarkers to detect skin disease or physiological conditions.

In this study, we aimed to identify novel biomarkers and to investigate the biochemical metabolic pathways using comparative metabolomic analysis of volar and non-volar skin tissues. Therefore, we performed a metabolomics analysis based on gas chromatography time-of-light mass spectrometry (GC/TOF-MS) data to determine the metabolite differences between volar and non-volar skin.

## 2. Results

### 2.1. Overview of Metabolite Profiles in Volar and Non-Volar Skin Tissue

GC-TOF/MS was used to analyze the derivative samples of volar and non-volar skin from the paws of mice. In total, 96 metabolites were identified after BinBase processing. These 96 metabolites were divided into different chemical categories based on their chemical structure, such as amino acids (25.00%), organic acids (19.80%), sugars and sugar alcohols (18.75%), amines (10.42%), fatty acids (12.50%), and phosphates (8.33%) (Table S1). These identified metabolites are involved in many important metabolic pathways in tissues, such as glucose, fructose-6-phosphate, phosphogluconic acid, and pyruvate in the glycolysis pathway; ketoglutaric acid and succinic acid in the tricarboxylic acid (TCA) cycle; leucine, lysine, methionine, and other amino acids in protein synthesis; nonanoate, palmitate, pentadecanoic acid, and stearic acid in fatty acid metabolism; and adenosine, ethanolamine, guanosine, and xanthine in nucleic acid metabolism. Changes in these key intracellular metabolic activities may affect the structural characteristics of skin. For example, changes in fatty acid metabolism pathways are known to affect keratinocyte renewal [23].

### 2.2. Principal Component Analysis (PCA) of Metabolite Profiles in Volar Skin and Non-Volar Skin Tissue

**Figure 1:**
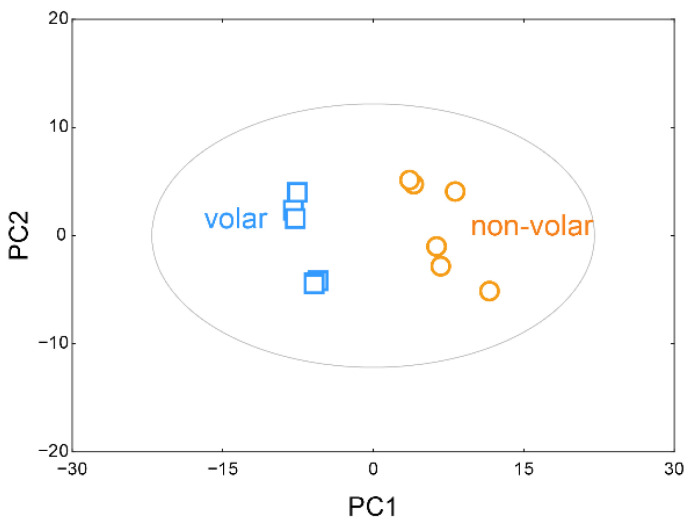
Principal component analysis (PCA) scores plot. PCA score plots based on GC/TOF-MS data show the relationship between volar and non-volar skin.

After normalizing the sum of the peak intensities of the identified metabolites using BinBase, multivariate statistical modeling was employed using PCA to find any natural groups in the data and outliers in the spectral data. As shown in Figure 1, metabolite-based PCA showed a very distinct difference in metabolite profiles between the volar and non-volar skin groups (R^2^X = 70.2%, Q^2^ = 78.4%).

A clear separation between volar skin and non-volar skin was seen by the 52.8% variance of the first principal component (PC1) and 17.3% variance of the second principal component (PC2). The selected 20 metabolites and their loading scores, which represent how each metabolite contributed to the new variables generated using the PCA model, are listed in Table 1. Of the identified 96 metabolites, 70 metabolites, including oxoproline, galactose, urea, palmitate, myo-inositol, glucose, lactic acid, mannitol, and stearic acid, positively contributed to PC1. In contrast, 26 metabolites, including ornithine, serine, glutamate, tyrosine, phenylalanine, succinic acid, and phosphate, contributed negatively to PC1.

### 2.3. Hierarchical Cluster Analysis (HCA) of Metabolites

**Figure 2:**
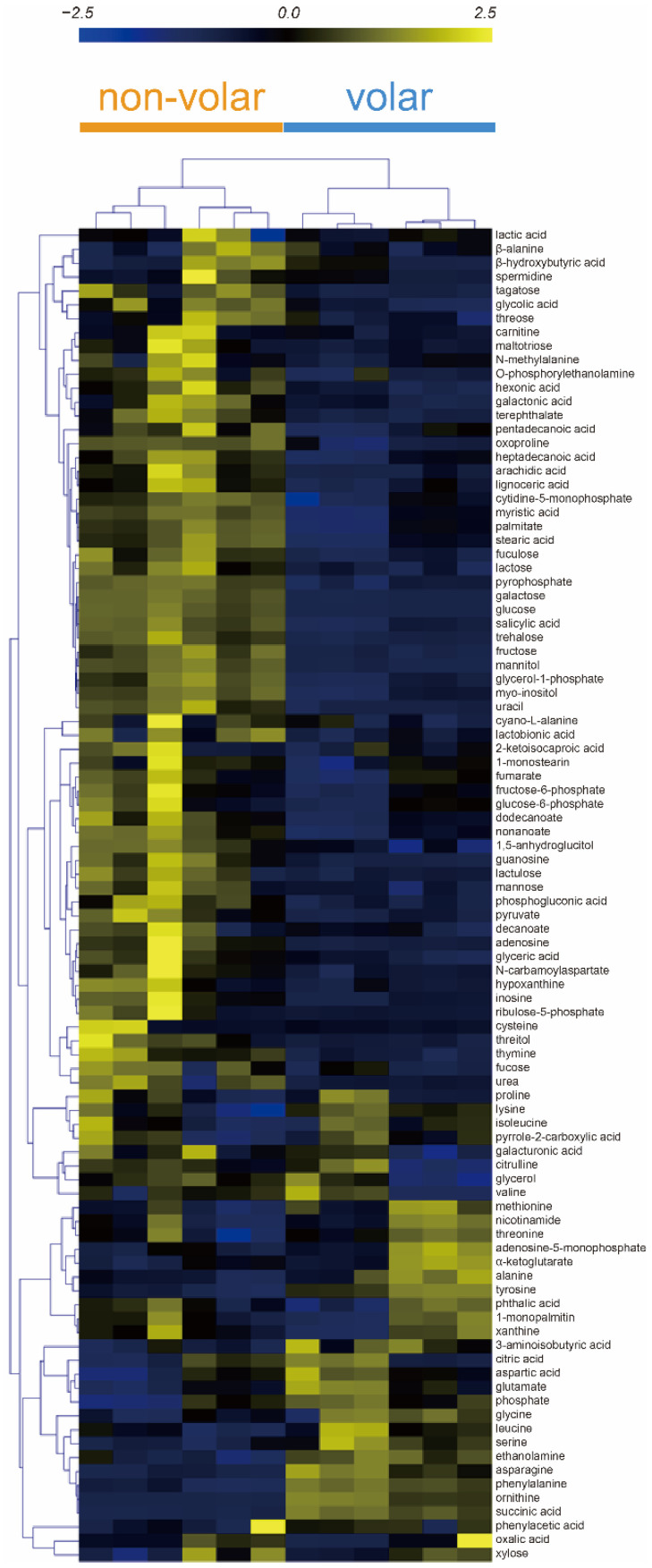
Hierarchical clustering analysis (HCA) of 96 metabolites from volar and non-volar skin tissue. Clustering similarity was evaluated based on the Pearson correlation coefficient and average linkage method. Each vertical column represents one of the six samples of volar skin and non-volar skin whereas each horizontal row represents a different metabolite.

The heatmap shows the metabolites and skin tissue for HCA. The HCA results showed that the metabolic patterns of the biological replicates were similar in volar and non-volar skin tissues. However, the metabolite profiles were clearly different between the two groups (Figure 2).

Metabolites such as asparagine, phenylalanine, succinic acid, ornithine, tyrosine, and ethanolamine were markedly increased in volar skin tissue, and myristic acid, stearic acid, palmitate, oxoproline, nonanoate, myo-inositol, pyrophosphate, gycerol-1-phosphate showed higher abundances in non-volar skin tissue. These results indicate that the distribution of metabolites in volar and non-volar skin differs significantly.

### 2.4. Screening of Metabolic Markers

When comparing the metabolite abundance differences between the volar and non-volar skin tissue groups, Student’s t-test was employed, and differentially identified metabolites were selected according to *p* < 0.05. The results showed that 60 compounds were significantly different between the two tissues (*p* < 0.05) (Table 2). The levels of 14 metabolites (ornithine, succinic acid, phenylalanine, asparagine, tyrosine, ethanolamine, serine, 3-aminoisobutyric acid, alanine, glutamate, methionine, aspartic acid, phosphate, and glycine) were significantly increased, whereas 46 metabolites, including galactose, glucose, mannitol, and fructose, were significantly decreased in volar skin compared with those in non-volar skin.

Orthogonal partial least squares discriminant analysis (orthoPLS-DA) was employed to calculate the variable importance in projection (VIP) scores between the volar and non-volar skin tissue groups; differentially identified metabolites were selected based on VIP scores > 1, and 50 metabolites were identified (Table 3).

In total, 50 features were screened as key markers for discrimination between volar and non-volar skin tissues following the rules of VIP > 1 and *p* < 0.05. Among these potential markers, nine metabolites (ornithine, succinic acid, phenylalanine, asparagine, tyrosine, ethanolamine, serine, 3-aminoisobutyric acid, and alanine) were higher in volar skin than in non-volar skin. However, the levels of 41 metabolites, such as galactose, glucose, mannitol, fructose, and trehalose, were significantly higher in the non-volar group.

### 2.5. Metabolic Pathway Analysis

**Figure 3:**
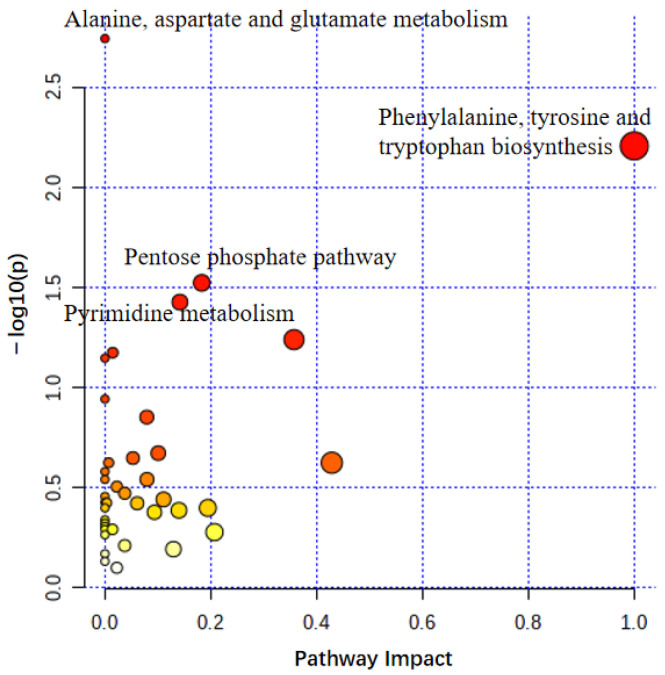
Use of MetaboAnalyst for summarizing pathway analysis. The color and size of the circles indicated their *p*-value and pathway impact value, respectively. The following showed significant differences between volar skin and non-volar skin at a significance level of *p* < 0.05: alanine, aspartate, and glutamate metabolism; phenylalanine, tyrosine, and tryptophan biosynthesis; pentose phosphate pathway; and pyrimidine metabolism.

Pathway analysis was performed using MetaboAnalyst5 (Figure 3) to investigate the metabolic pathways that differ depending on skin type. The *p*-value and pathway impact were calculated using enrichment analysis and pathway topology analysis, respectively. Four pathways were identified, namely, alanine, aspartate, and glutamate metabolism; phenylalanine, tyrosine, and tryptophan biosynthesis; pentose phosphate pathway; and pyrimidine metabolism—suggesting their clear association with the development of the volar skin phenotype.

## 3. Discussion

This is the first study to explore the differences in metabolite profiles between volar and non-volar skin tissues by metabolomic analysis. In total, 96 metabolites were identified through GC-TOF/MS and classified as sugar and sugar alcohols, amino acids, organic acids, amines, fatty acids, phosphates, and others based on their chemical structure. The metabolite profiles of volar and non-volar skin tissues were significantly discriminated using multivariate and cluster analysis. In particular, 50 of 96 metabolites showed significant differences between the two skin tissues types using Student’s *t*-test and VIP scores. These 50 metabolites were related to alanine, aspartate, and glutamate metabolism; phenylalanine, tyrosine, and tryptophan biosynthesis; pentose phosphate pathway; and pyrimidine metabolism. Moreover, a significant difference in the alanine, aspartate, and glutamate metabolic pathway was observed between volar skin and non-volar skin. In skin, keratinocytes and fibroblasts produce important proteins for maintaining their physiological functions and confer protection from dryness, damage, and infection. Previous studies indicate that the amino acid composition is important to restore collagen synthesis after UV irradiation; importantly, an unbalanced ratio of amino acids and proteins can cause critical dysfunctions, including loss of elasticity, dehydration, and skin thinning. Furthermore, a deficit in proteins in skin leads to severe skin diseases, such as eczemas, atopic dermatitis, and itching [24].

Impaired tryptophan transport can cause Hartnup disease, which is characterized by photosensitivity and a pellagra-like rash, suggesting that skin phenotype and function are related to tryptophan [24]. Further, the human skin contains enzymes that catalyze the conversion of tryptophan to melatonin, to protect the skin from oxidative stress [25]. Phenylalanine and tyrosine are important for melanin synthesis; phenylalanine is converted to tyrosine by the action of phenylalanine hydroxylase [26] and tyrosine is further oxidized to melanin by tyrosinase [27,28]. The amount and type of melanin determine the phenotype and color of skin, with darker skin having a higher melanin content than lighter skin. Therefore, the expression levels of phenylalanine and tyrosine can affect skin color. The present study revealed a significant difference in the phenylalanine, tyrosine, and tryptophan biosynthesis pathways in volar skin compared to those in non-volar skin, indicating a difference in melanin synthesis between the two different skin types, consistent with the fact that volar skin is lighter than non-volar skin.

The pentose phosphate pathway (PPP) supplies biosynthetic precursors for nucleotides and amino acids, maintains carbon homeostasis, provides reducing molecules for assimilation, and is critical for overcoming oxidative stress [29]. Glucose-6-phosphate dehydrogenase and glucose-1-phosphate dehydrogenase activities are higher in the palmar and plantar epidermis, human psoriatic epidermis, mouse tail scale epidermis, and keratinized zone pilaris, suggesting the involvement of PPP in these tissues [30]. In accordance with a previous study, this study showed perturbation of PPP in volar skin, suggesting that in the acanthotic epidermis, the citric acid cycle most likely shifts to PPP metabolism.

A fairly complete array of enzyme systems for the metabolism of purine, pyrimidine, and nucleosides has also been found in the human epidermis [31]. A large amount of nucleic acid synthesis may be required for continuous renewal of epidermal keratinocytes [32]. Further, in daily life, volar skin undergoes damage due to greater friction and mechanical shear force caused by grasping and other functions, compared to non-volar skin; the repair of these injuries also requires nucleic acid synthesis [33]. Taken together, it is reasonable to conclude that the pyrimidine metabolism pathway is significantly different between volar and non-volar skin types.

In summary, the present study demonstrated that volar and non-volar skin exhibit distinct metabolite profiles. Alteration in alanine, aspartate, and glutamate metabolism indicates that some amino acids enter the citric acid cycle through transamination and participate in energy metabolism; alteration in phenylalanine, tyrosine, and tryptophan biosynthesis explain the different colors in the two skin types. The pentose phosphate pathway and pyrimidine metabolism are significantly associated with the renewal of the outer layer of skin, and increased exposure to friction and stress in volar skin. Overall, these results provide valuable information to understand the differences between volar and non-volar skin and may contribute to both basic and translational studies for advancing knowledge and technology for site-specific skin identity studies.

## 4. Materials and Methods

### 4.1. Collection of Skin Tissue Samples

Skin tissue samples were collected from eight-week-old male and female C57BL/6 mice. Mouse forepaws were collected and cut carefully using scissors to separate the ventral (volar skin) and dorsal (non-volar skin) region. All tissues were stored at −80 °C for later use.

### 4.2. Preparation of Tissue Extracts for GC-TOF/MS

Metabolite extraction from skin tissues was performed as previously described [34]. Briefly, skin tissue was quickly mixed with 900 µL of a 1:2 MeOH/CHCl_3_ (1:2, *v*/*v*) solution. After the mixture was homogenized using an ultrasonic homogenizer for 5 min on ice, 120 µL of pre-cooled distilled H_2_O was added and then homogenized twice for 45 s with an interval of 45 s on ice. The samples were incubated at −20 °C for 1 h. To remove cells and other precipitated material, the samples were centrifuged at 14,000× *g* for 10 min at 4 °C. The polar upper phase was then collected and dried completely using a vacuum centrifugal concentrator (Micro-Cenvac NB-503CIR; N-Biotek, Bucheon, Korea) for derivatization. Next, 20 µL of 40 mg/mL methoxyamine hydrochloride (Sigma-Aldrich, Dorset, UK) dissolved in pyridine (Sigma-Aldrich, Bangalore, India) was added to the dried samples and mixed for 90 min at 30 °C; then, 45 µL of *N*-methyl-*N*-trimethylsilyl-trifluoroacetamide (MSTFA) (Sigma-Aldrich, Buchs, Switzerland) was added and incubated for 30 min at 37 °C. Finally, a mixture of fatty acid methyl esters (C8–C30) was added to the derivatized samples as an internal retention index.

### 4.3. GC-TOF/MS Analysis of Derivatized Samples

For metabolite analysis, an Agilent 7890B BC system (Agilent Technologies, Santa Clara, CA, USA) equipped with a Pegasus HT TOF mass spectrometer (LECO, St. Joseph, MI, USA) was used. An RTX-5Sil MS capillary column (30 m × 0.25 mm, 0.25 μm film thickness; Restek, Bellefonte, PA, USA), with an additional integrated guard column (10 m × 0.25 mm, 0.25 μm film thickness; Restek, Bellefonte, PA, USA) was injected with 1 µL of derivatives and subjected to an initial temperature of 50 °C with holding for 1 min, and then increased to 330 °C at 20 °C/min and held for 5 min. The injection temperature was 250 °C and the interface temperature was 280 °C. In the mass range of 85–500 *m*/*z*, mass spectra were collected by electron impact at 70 eV.

### 4.4. Statistical Analyses

Chroma TOF software (version 4.50; Leco, St. Joseph, MI, USA) was used to preprocess the GC/TOF-MS raw data; the preprocessed data were further processed using an in-house database, BinBase, as described previously [35,36]. Briefly, the algorithm of the BinBase database processes GC/TOF MS spectra data with criteria such as the retention index, unique mass, signal-to-noise (S/N) ratio, peak purity, and spectra similarity filters. The metabolites were identified with 80% occurrence in one or more classes of samples and known artifacts such as byproducts of derivatization reaction and polysiloxanes were systemically eliminated. The processed data were then normalized based on the sum of the peak intensities of the identified metabolites. For univariate and multivariate statistical analyses of the processed data, we used a principal component analysis (PCA) and orthogonal partial least squares discriminant analysis (orthoPLS-DA) with the variable importance in projection (VIP) value for each variable in the skin tissue samples, conducted using STATISTICA (version 7.1; StatSoft, Tulsa, OK, USA) and MetaboAnalyst (https://www.metaboanalyst.ca/; accessed on 2 June 2022) [37]. Hierarchical clustering analysis (HCA) and metabolic pathway analysis was performed using MeV (Dana-Farber Cancer Institute, Boston, MA, USA) [38] and MetaboAnalyst5.0, respectively.

## Figures and Tables

**Table 1:** Twenty metabolites with their high absolute loading scores on PC1 and PC2, as obtained by PCA.

PC1		PC2	
Metabolite	Loading Score	Metabolite	Loading Score
trehalose	0.990	β-hydroxybutyric acid	0.937
glucose	0.987	β-alanine	0.894
galactose	0.985	citric acid	0.794
salicylic acid	0.982	threose	0.727
pyrophosphate	0.975	spermidine	0.666
mannitol	0.969	glycolic acid	0.584
uracil	0.960	glycerol	0.572
myo-inositol	0.957	aspartic acid	0.561
glycerol-1-phosphate	0.952	phosphate	0.540
guanosine	0.948	valine	0.534
succinic acid	−0.958	nicotinamide	−0.896
ornithine	−0.946	threonine	−0.874
phenylalanine	−0.903	1-monopalmitin	−0.807
asparagine	−0.884	phthalic acid	−0.771
serine	−0.837	xanthine	−0.744
tyrosine	−0.826	glucose-6-phosphate	−0.696
ethanolamine	−0.821	methionine	−0.688
glutamate	−0.772	pyrrole-2-carboxylic acid	−0.665
phosphate	−0.725	2-ketoisocaproic acid	−0.633
aspartic acid	−0.665	lysine	−0.633

**Table 2:** Student’s t-test analysis of 96 metabolites from volar and non-volar skin tissues.

Metabolites	*p*-Value	Metabolites	*p*-Value	Metabolites	*p*-Value
ornithine	<0.001	threitol	0.001	ribulose-5-phosphate	0.059
succinic acid	<0.001	N-carbamoylaspartate	0.002	fumarate	0.059
glucose	<0.001	galactonic acid	0.002	adenosine-5-monophosphate	0.066
galactose	<0.001	lignoceric acid	0.002	α-ketoglutarate	0.077
mannitol	<0.001	dodecanoate	0.002	decanoate	0.078
trehalose	<0.001	stearic acid	0.002	lysine	0.079
fructose	<0.001	3-aminoisobutyric acid	0.003	cyano-L-alanine	0.080
adenosine	<0.001	glyceric acid	0.003	glucose-6-phosphate	0.088
phenylalanine	<0.001	myristic acid	0.004	N-methylalanine	0.093
asparagine	<0.001	1,5-anhydroglucitol	0.005	threonine	0.114
salicylic acid	<0.001	heptadecanoic acid	0.005	carnitine	0.123
uracil	<0.001	palmitate	0.006	nicotinamide	0.154
glycerol-1-phosphate	<0.001	ribulose-5-phosphate	0.007	glycerol	0.176
guanosine	<0.001	alanine	0.007	galacturonic acid	0.176
terephthalate	<0.001	glutamate	0.007	spermidine	0.180
tyrosine	<0.001	maltotriose	0.007	L-cysteine	0.184
pyrophosphate	<0.001	lactulose	0.008	2-ketoisocaproic acid	0.212
fuculose	<0.001	O-phosphorylethanolamine	0.008	β-hydroxybutyric acid	0.267
myo-inositol	<0.001	mannose	0.009	β-alanine	0.385
arachidic acid	<0.001	cytidine-5-monophosphate	0.009	isoleucine	0.418
phosphogluconic acid	<0.001	pentadecanoic acid	0.011	citrulline	0.430
pyruvate	<0.001	hypoxanthine	0.014	citric acid	0.440
ethanolamine	<0.001	fructose-6-phosphate	0.015	xanthine	0.529
thymine	<0.001	methionine	0.023	pyrrole-2-carboxylic acid	0.546
oxoproline	<0.001	aspartic acid	0.026	lactic acid	0.560
inosine	<0.001	phosphate	0.026	phenylacetic acid	0.678
hexonic acid	<0.001	glycine	0.028	phthalic acid	0.713
Serine	<0.001	lactobionic acid	0.030	oxalic acid	0.747
lactose	<0.001	maltotriose	0.056	1-monopalmitin	0.806
tagatose	<0.001	1-monostearin	0.056	valine	0.828
glycolic acid	<0.001	fucose	0.056	xylose	0.893
nonanoate	<0.001	urea	0.057	proline	0.924

**Table 3:** Metabolites screened from volar skin and non-volar skin.

Metabolites	*p*-Value	VIP Score	HMDB
Metabolites with higher intensity in volar than in non-volar skin			
ornithine	<0.001	1.385	HMDB0000214
succinic acid	<0.001	1.381	HMDB0000254
phenylalanine	<0.001	1.326	HMDB0000159
asparagine	<0.001	1.303	HMDB0000168
tyrosine	<0.001	1.293	HMDB0000158
ethanolamine	<0.001	1.249	HMDB0000149
serine	<0.001	1.224	HMDB0062263
3-aminoisobutyric acid	0.003	1.046	HMDB0003911
alanine	0.007	1.03	HMDB0000161
Metabolites with lower intensity in volar than in non-volar skin			
mannose	0.009	1.037	HMDB0000169
O-phosphorylethanolamine	0.008	1.035	HMDB0000224
lactulose	0.008	1.037	HMDB0000740
maltotriose	0.007	1.055	HMDB0001262
ribulose-5-phosphate	0.007	1.046	HMDB0000618
palmitate	0.006	1.014	HMDB0000220
heptadecanoic acid	0.005	1.044	HMDB0002259
1,5-anhydroglucitol	0.005	1.088	HMDB0002712
myristic acid	0.004	1.05	HMDB0000806
glyceric acid	0.003	1.114	HMDB0000139
stearic acid	0.002	1.091	HMDB0000827
dodecanoate	0.002	1.102	HMDB0000638
lignoceric acid	0.002	1.128	HMDB0002003
galactonic acid	0.002	1.167	HMDB0000565
N-carbamoylaspartate	0.002	1.148	HMDB0000828
threitol	0.001	1.142	HMDB0004136
nonanoate	<0.001	1.158	HMDB0000847
glycolic acid	<0.001	1.179	HMDB0000115
tagatose	<0.001	1.15	HMDB0003418
lactose	<0.001	1.218	HMDB0041627
hexonic acid	<0.001	1.254	HMDB0000625
inosine	<0.001	1.215	HMDB0000195
oxoproline	<0.001	1.219	HMDB0000267
thymine	<0.001	1.207	HMDB0000262
pyruvate	<0.001	1.243	HMDB0000243
phosphogluconic acid	<0.001	1.255	HMDB0001316
arachidic acid	<0.001	1.287	HMDB0002212
myo-inositol	<0.001	1.268	HMDB0000211
fuculose	<0.001	1.288	-
pyrophosphate	<0.001	1.276	HMDB0000250
terephthalate	<0.001	1.32	HMDB0002428
guanosine	<0.001	1.33	HMDB0000133
glycerol-1-phosphate	<0.001	1.322	HMDB0012208
uracil	<0.001	1.333	HMDB0000300
salicylic acid	<0.001	1.324	HMDB0001895
adenosine	<0.001	1.342	HMDB0000050
fructose	<0.001	1.367	HMDB0000660
trehalose	<0.001	1.361	HMDB0000975
mannitol	<0.001	1.393	HMDB0000765
galactose	<0.001	1.385	HMDB0000143
glucose	<0.001	1.385	HMDB0000122

## Data Availability

The data presented in this study are available on request from the corresponding author. The data are not publicly available due to a high level of privacy.

## Supplementary Materials

The following supporting information can be downloaded at: https://www.mdpi.com/article/10.3390/metabo12080717/s1, Table S1: Identified metabolites in volar skin and non-volar skin based on their chemical structures.
