# Supplementary material for: GC-TOF/MS-Based Metabolomics for Comparison of Volar and Non-Volar Skin Types
Source: Metabolites. 2022 Aug 3;12(8):717. doi: 10.3390/metabo12080717 (PMC9415232; doi:10.3390/metabo12080717)
Supplement: Supplementary file 1 [file metabolites-12-00717-s001.zip › metabolites-1836676-supplementary.pdf]

Supplemental material

Table S1. Identified metabolites in volar skin and non-volar skin based on their chemical structures.

**Identified metabolites**		
**Amines (10)**		
adenosine	inosine	uracil
ethanolamine	spermidine	hypoxanthine
guanosine	thymine	ornithine
xanthine		
**Amino acids (24)**		
2-ketoisocaproic acid	glutamate	N-methylalanine
alanine	glycine	oxoproline
asparagine	isoleucine	phenylalanine
aspartic acid	L-cysteine	proline
β-alanine	leucine	serine
citrulline	lysine	threonine
cyano-L-alanine	methionine	tyrosine
3-aminoisobutyric acid	N-carbamoylaspartate	valine
**Sugars and sugar alcohols (18)**		
1,5-anhydroglucitol	fuculose	maltotriose
glycerol	fructose	mannose
mannitol	galactose	tagatose
myo-inositol	glucose	threose
threitol	lactose	trehalose
fucose	lactulose	xylose
**Organic acids (19)**		
salicylic acid	glyceric acid	phosphogluconic acid
β-hydroxybutyric acid	glycolic acid	phthalic acid
citric acid	hexonic acid	α-ketoglutarate
fumarate	lactic acid	pyrrole-2-carboxylic acid
galactonic acid	oxalic acid	pyruvate
galacturonic acid	phenylacetic acid	succinic acid
terephthalate		
**Fatty acids (12)**		
1-monopalmitin	dodecanoate	lignoceric acid
1-monostearin	heptadecanoic acid	pentadecanoic acid
arachidic acid	nonanoate	palmitate
decanoate	myristic acid	stearic acid

**Phosphates (8)**		
adenosine-5-monophosphate	ribulose-5-phosphate	pyrophosphate
cytidine-5-monophosphate	glycerol-1-phosphate	phosphate
fructose-6-phosphate	glucose-6-phosphate	
**Others (5)**		
carnitine	urea	lactobionic acid
nicotinamide	O-phosphorylethanolamine	
